# The impact of normothermic and hypothermic preservation methods on kidney lipidome—comparative study using chemical biopsy with microextraction probes

**DOI:** 10.3389/fmolb.2024.1341108

**Published:** 2024-05-09

**Authors:** Natalia Warmuzińska, Kamil Łuczykowski, Iga Stryjak, Hernando Rosales-Solano, Peter Urbanellis, Janusz Pawliszyn, Markus Selzner, Barbara Bojko

**Affiliations:** ^1^ Department of Pharmacodynamics and Molecular Pharmacology, Faculty of Pharmacy, Nicolaus Copernicus University in Torun, Collegium Medicum in Bydgoszcz, Bydgoszcz, Poland; ^2^ Department of Chemistry, University of Waterloo, Waterloo, ON, Canada; ^3^ Ajmera Transplant Center, Department of Surgery, Toronto General Hospital, University Health Network, Toronto, ON, Canada; ^4^ Department of Medicine, Toronto General Hospital, Toronto, ON, Canada

**Keywords:** solid-phase microextraction, SPME, LC-MS, kidney transplantation, lipidomics, graft quality assessment, kidney perfusion

## Abstract

**Introduction:**

Normothermic *ex vivo* kidney perfusion (NEVKP) is designed to replicate physiological conditions to improve graft outcomes. A comparison of the impact of hypothermic and normothermic preservation techniques on graft quality was performed by lipidomic profiling using solid-phase microextraction (SPME) chemical biopsy as a minimally invasive sampling approach.

**Methods:**

Direct kidney sampling was conducted using SPME probes coated with a mixed-mode extraction phase in a porcine autotransplantation model of the renal donor after cardiac death, comparing three preservation methods: static cold storage (SCS), NEVKP, and hypothermic machine perfusion (HMP). The lipidomic analysis was done using ultra-high-performance liquid chromatography coupled with a Q-Exactive Focus Orbitrap mass spectrometer.

**Results:**

Chemometric analysis showed that the NEVLP group was separated from SCS and HMP groups. Further in-depth analyses indicated significantly (*p* < 0.05, VIP > 1) higher levels of acylcarnitines, phosphocholines, ether-linked and longer-chain phosphoethanolamines, triacylglycerols and most lysophosphocholines and lysophosphoethanolamines in the hypothermic preservation group. The results showed that the preservation temperature has a more significant impact on the lipidomic profile of the kidney than the preservation method’s mechanical characteristics.

**Conclusion:**

Higher levels of lipids detected in the hypothermic preservation group may be related to ischemia-reperfusion injury, mitochondrial dysfunction, pro-inflammatory effect, and oxidative stress. Obtained results suggest the NEVKP method’s beneficial effect on graft function and confirm that SPME chemical biopsy enables low-invasive and repeated sampling of the same tissue, allowing tracking alterations in the graft throughout the entire transplantation procedure.

## 1 Introduction

Kidney transplantation is a life-saving method for patients with end-stage renal dysfunction that enables higher survival rates and patient quality of life compared to dialysis treatment. Unfortunately, an ongoing organ shortage has led to the rapid growth in the number of patients on kidney waiting lists (Swanson et al., 2020; Warmuzińska et al., 2022). This growing gap in supply and demand has led clinicians to explore the possibility of using kidneys recovered from extended criteria donors (ECD) or donation after circulatory death (DCD) donors; however, recipients of ECD kidney grafts tend to have worse outcomes than those receiving organs from standard criteria donors, including being at a higher risk of delayed graft function (DGF) and primary nonfunction incidence (Urbanellis et al., 2020; Warmuzińska et al., 2022). Hence, strategies for reducing preservation injury and monitoring graft function are of intense interest. At present, static cold storage (SCS) and hypothermic machine perfusion (HMP) are the most common preservation methods applied in clinical settings. SCS involves submerging the kidney in a cold preservation fluid and then placing it on ice in an icebox; in contrast, HMP entails using a device to pump cold preservation fluid through the renal vasculature, which has been demonstrated to be more effective at preserving marginal and DCD grafts compared to SCS (Lindell et al., 2013; Urbanellis et al., 2020; Warmuzińska et al., 2022). Normothermic *ex vivo* kidney perfusion (NEVKP) is a novel dynamic preservation strategy applying a perfusion solution’s circulation through the kidney. NEVKP conditions are developed to replicate physiological conditions as closely as possible to reduce cold ischemia damage and improve graft outcome (Resch et al., 2020; Hosgood et al., 2021). While several studies have attained promising results indicating NEVKP’s superiority over SCS, this application is still in the experimental stage (Kaths et al., 2017; Urbanellis et al., 2020). One notable problem is the need for more accurate methods of evaluating graft quality and assessing donor risk, especially concerning marginal grafts. A kidney’s suitability for transplantation is determined based on detailed parameters, including the donor’s medical history, visual assessment, and examination results (Warmuzińska et al., 2022). The visual evaluation of donor organs is frequently fundamental in decision-making. However, although macroscopic inspection can help diagnose tumors and anatomical changes, this method is subjective and depends on the transplant team’s experience level (Dare et al., 2014). Consequently, pretransplant biopsies remain the gold standard for identifying donor kidney injury. Histological examinations are often applied selectively, mainly in marginal grafts, and the frequency of performed biopsies varies between medical facilities and countries (Dare et al., 2014; Moeckli et al., 2019; Warmuzińska et al., 2022). Moreover, the use of biopsies is hampered by two significant limitations: the low reproducibility of results between on-call pathologists and their time-consuming nature (Azancot et al., 2014). Additionally, the number of allowable biopsies during the transplantation procedure is usually restricted to a single sampling due to their invasiveness, which limits their application for capturing dynamic changes and time-series analyses (Bojko, 2022). Therefore, new representative organ-quality assessment methods are needed to increase the number of organs available for transplantation. In this study, we assess the viability of solid-phase microextraction (SPME) chemical biopsy as a method for evaluating the impact of hypothermic and normothermic preservation techniques on the lipidomic profile of the kidney. The small diameter of the SPME probe (∼200 μm) enables minimal invasiveness and allows for several samplings of the same organ without damaging the tissue. Furthermore, SPME combines sampling, extraction, and metabolite quenching into a single step, which makes it a valuable tool for on-site analysis. Finally, SPME’s low invasiveness enables its application for monitoring of changes in the organ throughout the entire transplantation procedure, beginning with its removal from the donor’s body, through its preservation, and ending with its revascularization in the recipient’s body (Łuczykowski et al., 2023).

## 2 Materials and methods

### 2.1 Animals

Eight 3-month-old male Yorkshire pigs (≈30 kg) were housed for 1 week prior to the experiments, with water and food being provided *ad libitum*. All animals received humane care in compliance with the “Principles of Laboratory Animal Care” formulated by the National Society for Medical Research and the “Guide for the Care of Laboratory Animals” published by the National Institutes of Health and the ARRIVE guidelines 2.0. The study protocol was approved by the Animal Care Committee at the Toronto General Research Institute, Ontario, Canada.

### 2.2 Study design

**FIGURE 1:**
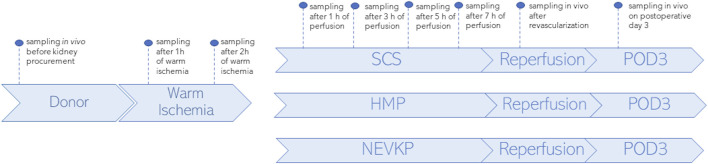
Study design. SPME sampling was performed *in vivo* prior to kidney procurement; after 1 h and 2 h of warm ischemia; after 1 h, 3 h, 5 h, and 7 h of perfusion; *in vivo* immediately after revascularization (reperfusion), and *in vivo* under deep anesthesia at the time of sacrifice on postoperative day 3 (POD3). Three types of kidney preservation methods—SCS, HMP, and NEVKP—were compared in the DCD porcine model of renal autotransplantation.

SPME fibers coated with a mixed-mode extraction phase (coating length: 7 mm) were applied for direct kidney sampling in three porcine models of renal DCD autotransplantation using different preservation methods: an 8-h SCS group (*n* = 3), an 8-h NEVKP group (*n* = 3), and an 8-h HMP group (*n* = 2). The autotransplantation and anesthetic procedures, warm ischemia induction, and NEVKP, HMP, and SCS conditions are described elsewhere (Kaths et al., 2015; Kaths et al., 2016; Kaths et al., 2018; Urbanellis et al., 2020). SPME sampling was performed *in vivo* prior to kidney procurement; after 1 h and 2 h of warm ischemia; after 1 h, 3 h, 5 h, and 7 h of perfusion; *in vivo* immediately after revascularization (reperfusion), and *in vivo* under deep anesthesia at the time of sacrifice on postoperative day 3 (POD3). The study protocol is illustrated in Figure 1. Before sampling, all fibers were preconditioned for 60 min in a methanol/water (50:50 v/v) solution, followed by rinsing with purified water for a few seconds. The extractions were performed by inserting the SPME fiber into the kidney cortex for 30 min at each time point. After sampling, the fibers were removed from the organ, quickly rinsed with water, and then gently dried with kimwipes to remove any tissue or blood residue. Next, the fibers were placed into empty glass vials and stored in a freezer at −80°C until analysis. All fibers were desorbed immediately before instrumental analysis. For desorption, the fibers were inserted into 200 μL of isopropanol:methanol (1:1 v/v) solution with the use of silanized inserts and agitated (1,200 rpm) using a BenchMixer™ MultiTube Vortexer (Benchmark Scientific, Edison, United States) for 120 min. After desorption, extracts were ready for instrumental analysis. The extraction blanks consisted of fibers that were prepared using the same protocol as the rest of the fibers, but with the extraction step being omitted.

### 2.3 Liquid chromatography–high resolution mass spectrometry analysis (LC-HRMS)

Untargeted lipidomics analysis was performed using an LC-HRMS procedure based on the coupling of an ultra-high performance liquid chromatograph and a Q-Exactive Focus Orbitrap mass spectrometer. Data acquisition was performed using dedicated Thermo Scientific software, namely, Xcalibur 4.2 and Free Style 1.4 (Thermo Fisher Scientific, San Jose, California, United States). The instrument was calibrated by injecting calibrants every 72 h, resulting in a mass accuracy of <2 ppm. During analysis, the samples were randomized and pooled quality control (QC) samples containing 10 μL of each sample were run every 8–10 injections to monitor instrument performance and analyte stability. Chromatographic separation was carried out on a hydrophilic interaction liquid chromatography (HILIC) column (SeQuant ZIC-cHILIC, 3 μm, 100 mm × 2.1 mm) and in reversed-phase (RP) using a C18 column (Waters, XSelect CSH C18, 3.5 µm, 2.1 mm × 75 mm) to cover a wide range of lipids. The mobile phases for the HILIC column were 5 mM ammonium acetate in water (A) and acetonitrile (B). The mobile phases for the RP column were: phase A consisted of water: methanol (60:40; v/v) and phase B of isopropanol: methanol (90:10; v/v), both containing 10 mM ammonium acetate and 1 mM acetic acid. The gradients were as described previously (Stryjak et al., 2020). The analyses were performed in positive and negative electrospray ionization mode. For positive ionization mode, the mass spectrometer parameters for the HILIC separations were as follows: a spray voltage of 1,500V; a capillary temperature of 325°C; sheath gas at 60 a.u.; an aux gas flow rate of 30 a.u.; a spare gas flow rate of 2 a.u.; a probe heater temperature of 325°C; an S-Lens radio frequency level of 55%; an S-lens voltage of 25 V; and a skimmer voltage of 15 V. For the RP analysis, the following parameters were employed: a spray voltage of 3500V; a capillary temperature of 275°C; sheath gas at 20 a.u.; an aux gas flow rate of 10 a.u.; a spare gas flow rate of 2 a.u.; a probe heater temperature of 300 °C; an S-Lens radio frequency level of 55%; an S-lens voltage of 25 V; and a skimmer voltage of 15 V. For negative ionization mode, the mass spectrometer parameters for the HILIC separations were as follows: a spray voltage of 1300V; a capillary temperature of 263°C; sheath gas at 60 a.u.; an aux gas flow rate of 30 a.u.; a spare gas flow rate of 2 a.u.; a probe heater temperature of 425°C; an S-Lens radio frequency level of 55%; an S-lens voltage of 25 V; and a skimmer voltage of 15 V. For the RP analysis, the following HESI ion source parameters were employed: a spray voltage of 3,500V; a capillary temperature of 275°C; sheath gas at 30 a.u.; an aux gas flow rate of 10 a.u.; a spare gas flow rate of 2 a.u.; a probe heater temperature of 300°C; an S-Lens radio frequency level of 55%; an S-lens voltage of 25 V; and a skimmer voltage of 15 V. The putative identification of compounds was confirmed in Full MS/dd-MS2 mode using the following fragmentation parameters: mass resolution—35,000 full width at half maximum (FWHM); AGC target—2E4; minimum AGC—8E3; intensity threshold—auto; maximum IT—auto; isolation window—3.0 *m*/*z*; stepped collision energy—20 V, 30 V, 50 V; loop count—2; dynamic exclusion—auto.

### 2.4 Data processing and statistical analysis

Raw data from each LC-HRMS analysis were processed independently using LipidSearch 4.1.30 (Thermo Fisher Scientific, San Jose, California, United States) software with the following parameters: peak intensity >10,000; a precursor tolerance of 5 ppm; a product tolerance of 10 ppm; an m-score threshold of 2; a Quan *m/z* tolerance of ±5 ppm; a Quan RT (retention time) range of 0.5 min; and the use of a main isomer filter. H^+^, NH4^+^, and Na^+^ adducts were considered in positive ion mode, while H^−^ and ^+^CH_3_COO were considered in negative ion mode. After completing the lipid identification step, the alignment process was performed using the LipidSearch software with the following parameters: an m-Score threshold of 10; a retention time tolerance of 0.25 min; a QC-to-extraction-blank ratio of >5; and a max 30% RSD in the QC. The search mode function of the software sought matches of parent peaks (full scan MS) and product peaks (fragments, MS/MS) with the lipid database entries. The software assigns four grades of identification of decreasing quality (A–D) to each feature. Identification grade filtering was applied to filter false positive lipid ID from LipidSearch results. Only lipids species with grades A and B were considered in further analyses. Grade A indicates that both lipid class and all fatty acid chains belonging to a given lipid were completely identified; grade B indicates full identification of lipid class and partial identification of fatty acid chains. The peak areas for the obtained compounds were analyzed using MetaboAnalyst 4.0 and Statistica 13.3 PL software (StatSoft, Inc., Tulsa, Oklahoma, United States). All missing values were replaced with small values that were assumed to be a detection limit. A UpSet plot was made with the lists of compounds annotated in the different LC-HRMS analyses using the UpSet plot generator tool (https://www.chiplot.online/upset_plot.html) to evaluate the number of lipids species identified in each analytical block. Data were normalized by median, log-transformation, and Pareto scaling, and statistical significance was calculated based on the Kruskal-Wallis test and the Mann–Whitney U test with FDR correction. A *post hoc* test with multiple comparisons of mean ranks for all groups with a Bonferroni correction followed the Kruskal-Wallis test. A *p*-value of <0.05 was considered significant. In addition, principal component analysis (PCA) and partial least squares discriminant analysis (PLS-DA) were conducted to visually assess the separation between sample groups, with variable importance in projection (VIP) scores >1 being used as a criterion for detecting the relevant variables in the context of the model’s predictive capability. Each model was validated via Leave-one-out cross-validation and refined with a permutation test. The model was considered to have passed permutation when the *p*-value was lower than 0.05. The Friedman test was employed to search for compounds with relative concentrations that changed throughout perfusion (across specific time points). After statistical analysis, the results from each LC-HRMS block were combined in tables to increase the clarity of the results. If a compound was considered statistically significant from more than one analytical condition, it was placed in tables only once with the most significant *p*-value to avoid duplication of information.

## 3 Results

**FIGURE 2:**
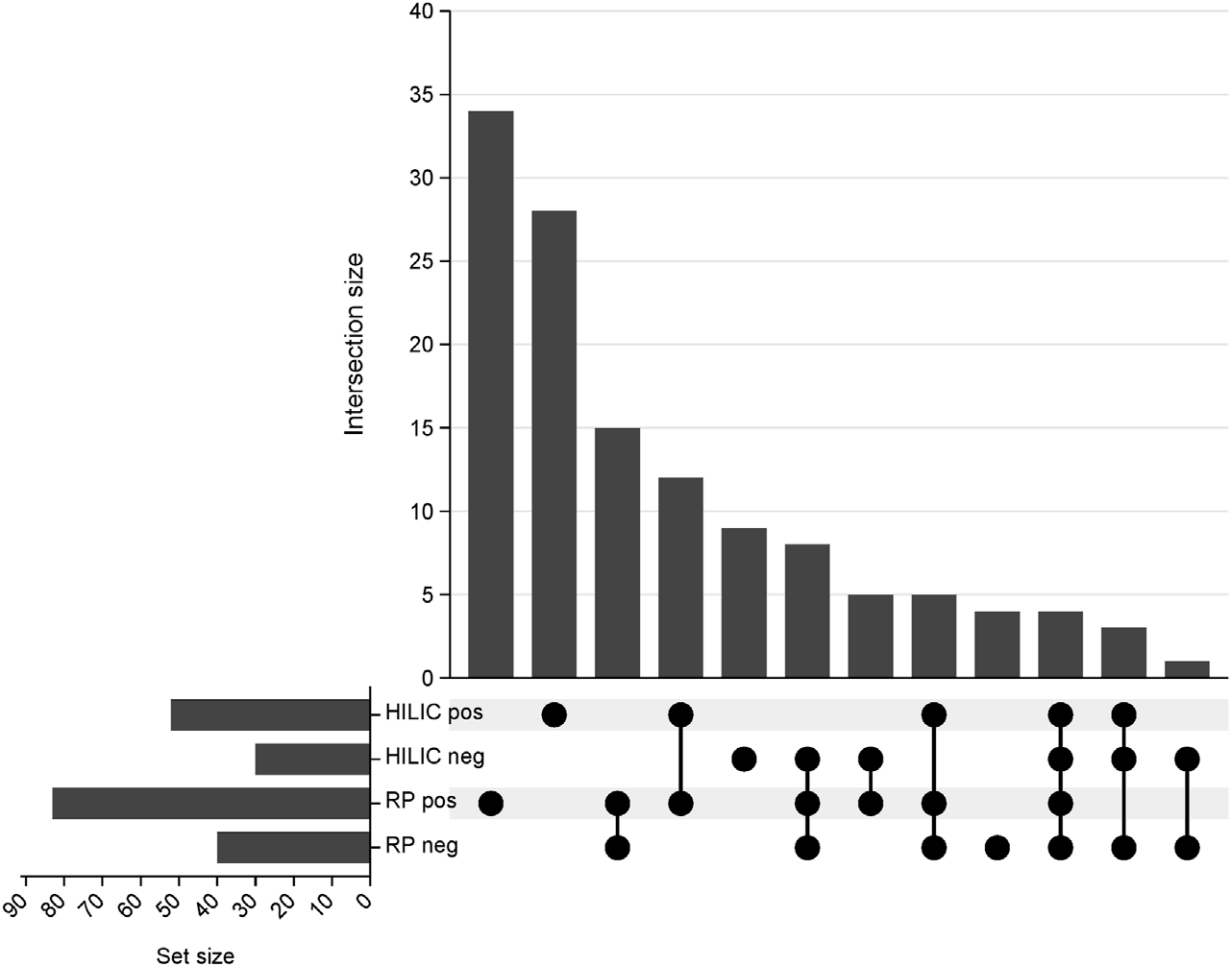
UpSet plot representing the number of compounds annotated in each analytical block. RP pos- reversed-phase in positive ionization mode; RP neg- reversed-phase in negative ionization mode; HILIC pos- hydrophilic interaction liquid chromatography in positive ionization; HILIC neg- hydrophilic interaction liquid chromatography in negative ionization mode.

The proposed method was employed to investigate changes in the lipidomic profiles of kidneys during transplantation and preservation. Principal component analysis was employed to confirm the quality of the instrumental analysis for all combinations of chromatographic separation and ionization mode. As shown in Supplementary Figure S1, the pooled QC samples formed a tight cluster, thus confirming the good quality of the analytical performance. Using all four blocks of LC-HRMS analysis, 128 lipid species belonging to 14 lipid classes were annotated with level 2 confidence in metabolomics compound identification. A UpSet plot representing the number of compounds annotated in each analytical block is shown in Figure 2. A list of the identified lipid species is provided in Supplementary Table S1.

The analysis of the results was divided into four parts: 1) analysis of how warm ischemia influenced the lipidomic profiles of the kidneys; 2) comparison of the three organ-preservation methods; 3) monitoring changes across time; and 4) investigation of the influence of transplantation procedure on kidney grafts.

### 3.1 Influence of warm ischemia on kidney lipidomic profiles

**FIGURE 3:**
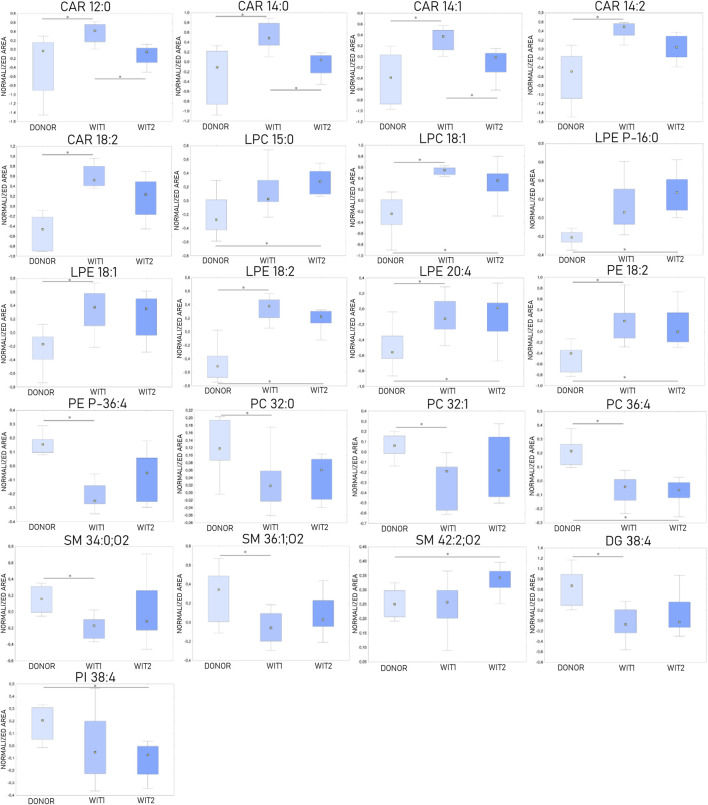
Changes in levels of selected lipids during warm ischemia (*n* = 8). The boxplots display normalized peak areas. The rectangle’s height represents the normalized peak areas in the interquartile range (Q1 and Q3). The upper whisker denotes the largest data point (excluding any outliers), while the lower whisker denotes the lowest data point (excluding any outliers). The median normalized peak area of each group is indicated with a yellow square. * is a *p*-value < 0.05. WIT1—1 h of warm ischemia; WIT2—2 h of warm ischemia.

The Kruskal-Wallis test, followed by *post hoc* test, was used to identify changes that occurred during warm ischemia. The results indicated that discriminative changes were mostly visible at the first sampling point of warm ischemia time (WIT). Among the identified lipids, an increase in acylcarnitines (CARs), lysophosphocholines (LPCs), and lysophosphoethanolamines (LPEs) was observed after 45 min of warm ischemia, while a corresponding decrease in phosphocholines (PCs) and sphingomyelins (SMs) was also noted. Boxplots of statistically significant lipids are shown in Figure 3.

### 3.2 Comparison of different kidney preservation methods

**FIGURE 4:**
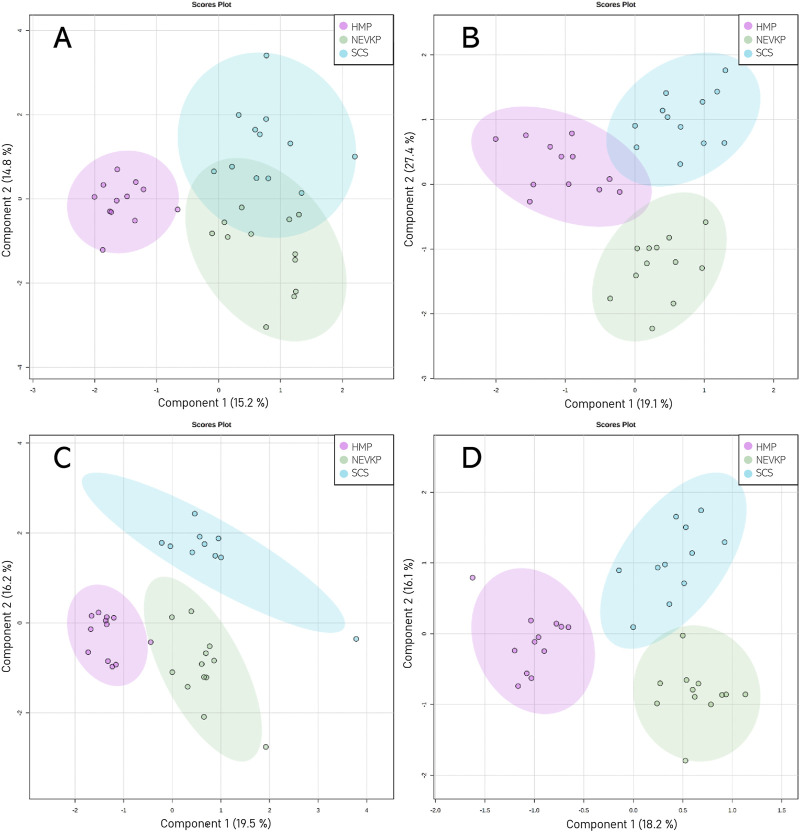
Score plots (PLS-DA) showing separation based on different types of kidney preservation. HILIC analyses in **(A)** positive and **(B)** negative ionization modes and RP analyses in **(C)** positive and **(D)** negative ionization modes. For HILIC analyses, the Q2 and R2 values for the PLS-DA model were 71% and 88% for positive ionization mode and 57% and 74% for negative ionization mode, respectively. For the RP analyses, the Q2 and R2 values for the PLS-DA model were 76% and 92% for positive ionization mode and 59% and 87% for negative ionization mode, respectively.

Chemometric analysis was conducted to visualize the data and investigate the differences in the kidney lipidomic profiles in the SCS, NEVKP, and HMP groups. The two-dimensional scoring plots (PC1 vs. PC2) presented in Supplementary Figure S2 revealed major differences in the lipidomic patterns of samples harvested under different preservation conditions. In all analyses, the lipidomes of the renal tissue from the NEVLP group showed clear separation from those of the SCS and HMP groups. In contrast, the data points in the scoring plots for the SCS and HMP groups had slightly overlapping distributions (Supplementary Figure S2). PLS-DA was applied to more accurately model differences in the lipidomic profiles of the kidneys in each preservation method group (Figure 4). Each model was validated via leave-one-out cross-validation and refined using a permutation test (permutation number = 1,000), which yielded significant (*p* < 0.05) quality parameters. This statistical analysis produced a set of compounds that successfully differentiated the different types of kidney preservation. A VIP score value > 1 was selected as a cut-off value. Furthermore, the Kruskal-Wallis test (*p* < 0.05) was used to compare the NEVKP, HMP, and SCS groups, while the discriminative compounds were selected based on chemometric and univariate analysis. Supplementary Table S2 lists the compounds meeting the above-mentioned criteria.

**FIGURE 5:**
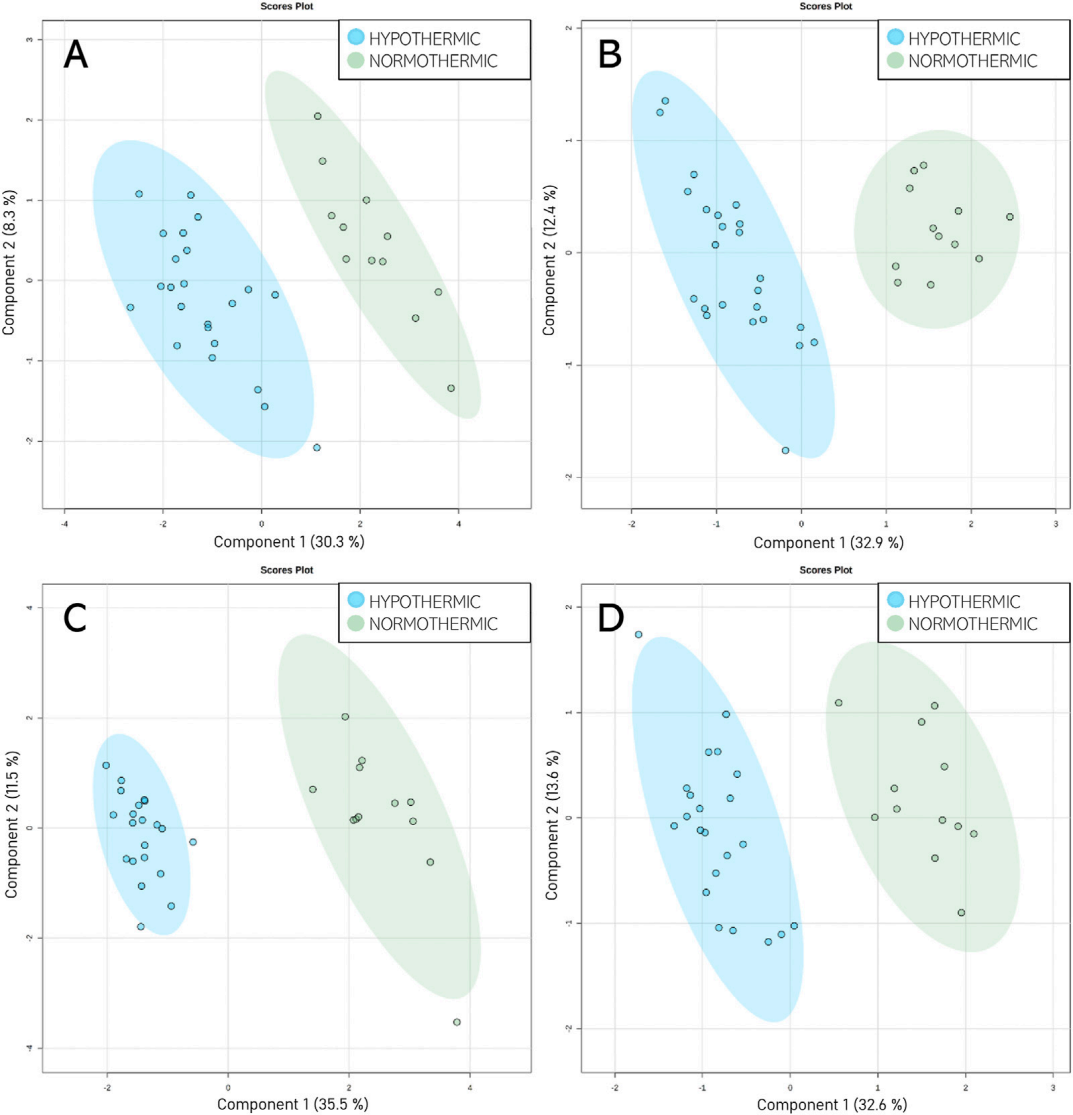
Score plots (PLS-DA) showing separation between the normothermic and hypothermic preservation methods. HILIC analyses in **(A)** positive and **(B)** negative ionization modes and RP analyses in **(C)** positive and **(D)** negative ionization modes. For HILIC analyses, the Q2 and R2 values for the PLS-DA model were 84% and 95% for positive ionization mode and 88% and 95% for negative ionization mode, respectively. For the RP analyses, the Q2 and R2 values for the PLS-DA model were 94% and 97% for positive ionization mode and 88% and 95% for negative ionization mode, respectively.

**FIGURE 6:**
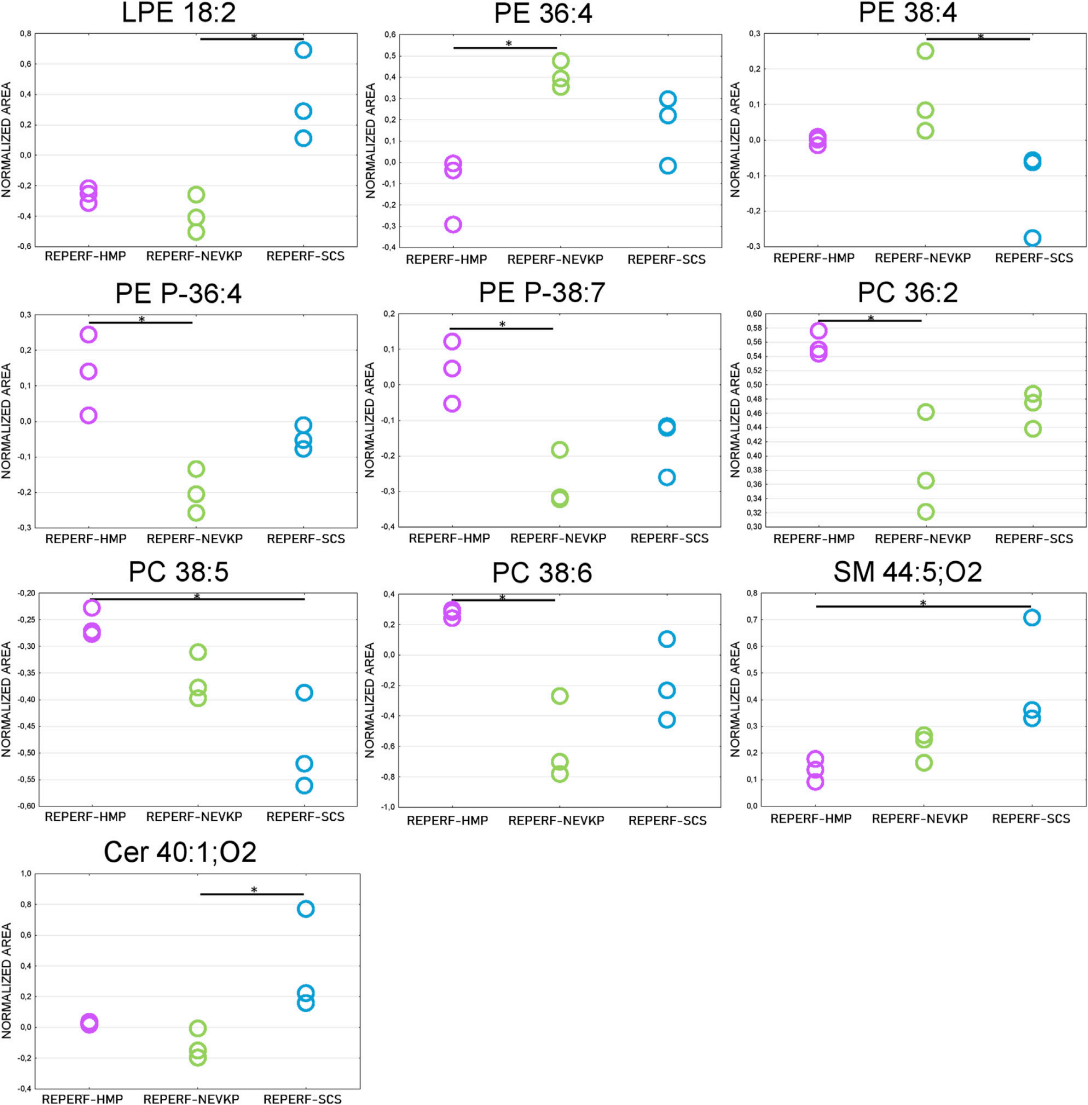
Differences in levels of selected compounds detected at reperfusion between the three preservation groups. * is a *p*-value < 0.05. NEVKP = green; HMP = pink; SCS = blue.

Given that unsupervised analysis indicated that the observed differences were mainly related to the preservation temperature, additional PCA (Supplementary Figure S3) and PLS-DA (Figure 5) analyses were conducted to identify the compounds that statistically differentiated the hypothermic and normothermic preservation methods. These analyses were confirmed via leave-one-out cross-validation and a positive permutation test (permutation number = 1,000; *p* < 0.05). In addition, the Mann–Whitney U test with FDR correction was also applied to select statistically important compounds. Supplementary Table S3 lists the compounds with a *p*-value < 0.05 and/or a VIP value > 1. Moreover, samples collected from all preservation groups after reperfusion were compared to identify differences occurring in kidney tissue *in vivo* after using a given preservation method. Strip plots of statistically significant compounds are shown in Figure 6. After reperfusion, significantly fewer differentiating compounds were identified than during preservation. A similar comparison was performed for samples collected on postoperative day 3. However, this comparison was possible only for the samples from the NEVKP and SCS groups, as the POD3 samples from the HMP group were rendered unrepresentative due to damage, and therefore had to be omitted. Nonetheless, a comparison of samples collected from the NEVKP and SCS groups at POD3 did not reveal any significant differentiating lipids.

### 3.3 Changes across time

**FIGURE 7:**
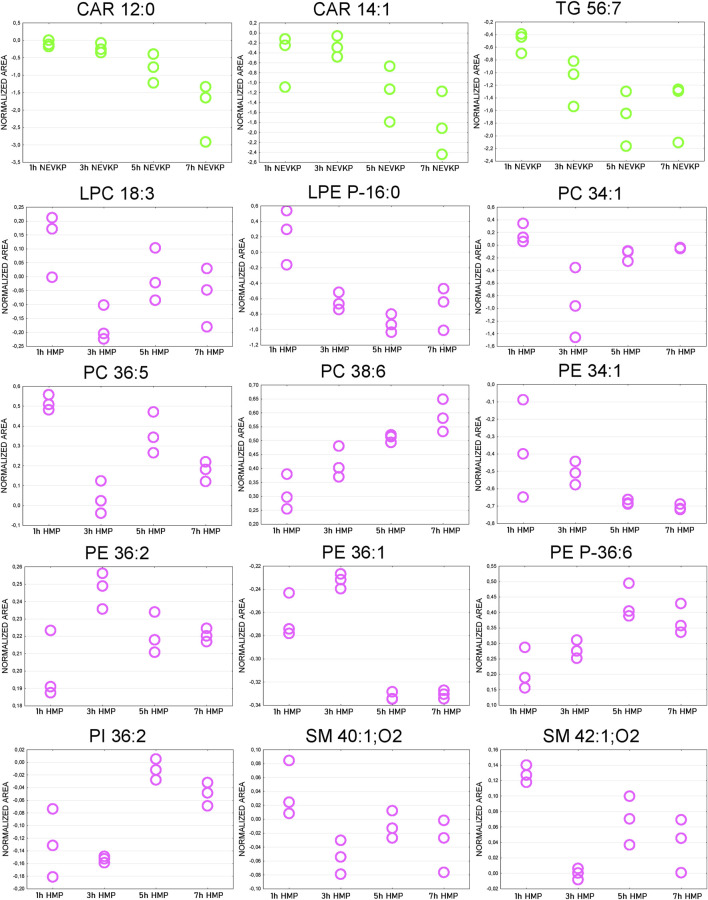
Strip plots of selected statistically significant changes between lipids and time points throughout kidney perfusion. NEVKP = green; HMP = pink.

The Friedman test was used to evaluate changes throughout perfusion. The majority of statistically significant changes in lipids levels were found in the samples collected during mechanical perfusion (especially HMP). For NEVKP, a decrease was observed for CARs (CAR 12:0, CAR 14:1) and triacylglycerol (TG) 56:7. For HMP samples, changes throughout perfusion were mainly observed for PCs, phosphoethanolamines (PEs), and SMs but no dominant trend was observed. Strip plots of the selected compounds are shown in Figure 7, and a list of statistically significant compounds is provided in Supplementary Table S4.

Next, differences between the reperfusion and POD3 samples were evaluated. As mentioned before, the POD3 samples from the HMP group were removed from the analysis; hence, comparisons were performed only for the samples from the NEVKP and SCS groups. The analysis revealed more differentiating lipids in the SCS group (21 compounds) than in the NEVKP group (3 compounds). In the NEVKP group, all statistically significant lipids were present at higher levels on POD3 than after reperfusion. In the SCS group, elevated levels of ether-linked phospholipids and PCs with 32 carbon chains were observed on POD3, along with a corresponding reduction in SMs, PEs, PC 35:6, and PC 38:5. A list of statistically significant compounds is shown in Supplementary Table S5.

### 3.4 Influence of transplantation procedure on kidney grafts

A Mann-Whitney U test with FDR correction was carried out to determine how the transplantation and preservation procedures affected the lipidomic profiles of the kidneys. For the SCS and NEVKP groups, comparisons of lipidomic profiles at donation and reperfusion and donation and POD3 were performed. For the HMP group, a comparison at donation and reperfusion was conducted. As shown in Supplementary Table S6, most of the statistically significant compounds in the SCS group were found in the donor and POD3 comparison. Conversely, in the comparison of donors and reperfusion, most of the significantly differentiating compounds were found in the NEVKP group; however, most of these compounds were present at lower levels after reperfusion. While the comparison of donors and POD 3 revealed similar change trends for the SCS and NEVKP groups, the alterations were usually more noticeable in the SCS group, as evidenced by the fold change. On POD3, both groups exhibited elevated levels CARs, LPEs, and PCs and PEs (including ether-linked) with relatively shorter chains, along with a corresponding in PCs with longer chains. Additionally, a reduction in PSs, SMs, and PEs with longer chains was also observed in the SCS group.

## 4 Discussion

Organ-preservation methods have garnered significant interest in graft quality evaluation, advanced organ monitoring, and the treatment of transplanted kidneys during machine perfusion. This study further explores these trends and, to the best of our knowledge, it is the first to apply SPME to compare SCS, NEVKP, and HMP preservation methods in a porcine DCD autotransplantation model. Moreover, this study highlights the alterations in the lipidomic profile during warm ischemia and perfusion, as well as after transplantation. The minimally invasive SPME chemical biopsy allows for the repeated direct sampling of the organ, as it does not require any tissue collection. Thanks to this undisputed advantage, it was possible to validate the PLS-DA models and identify significantly differentiating lipids using a relatively small number of animals. Additionally, this study employed four LC-HRMS analyses to cover a wide range of lipids. The analysis showed that this approach allowed the identification of more lipids than was possible using only one type of chromatographic separation. The largest number of unique compounds were identified with the use of RP and HILIC separation in positive ionization mode. In negative ionization mode, using HILIC and RP separations, 9 and 4 unique compounds were identified, respectively. Therefore, based on the results obtained and taking into account the time-consuming nature of the analyses, the consumption of organic reagents, the amount of data obtained, and the benefits of using additional LC-HRMS analyses in future studies, it is worth considering limiting the number of analyzes used, thus choosing the best compromise between advantages and disadvantages.

A thorough analysis of kidney graft tissue during warm ischemia indicated increased levels of CARs, LPCs, and LPEs. CARs are established mitochondrial biomarkers in neonatal screening; however, they are not routinely used beyond this screening, despite the growing evidence of their biomarker potential among disorders such as diabetes, sepsis, cancer, and heart failure (Breit and Weinberger, 2016; McCann et al., 2021). Moreover, alterations of CARs related to kidney disease, including acute kidney injury, progression of chronic kidney disease (CKD), and diabetic nephropathy, have been previously reported in several reports (Breit and Weinberger, 2016; Hocher and Adamski, 2017; Afshinnia et al., 2018; Kordalewska et al., 2019; Andrianova et al., 2020). In this study, significantly elevated levels of four long-chain CARs (CAR 14:0, CAR 14:1, CAR 14:2, CAR 18:2) and one medium-chain CAR (CAR 12:0) were mainly observed after 45 min of warm ischemia. CARs are crucial for transporting long-chain fatty acids into the mitochondria to ensure the β-oxidation process (Breit and Weinberger, 2016; Kordalewska et al., 2019). Thus, the increase of CARs observed during warm ischemia may be related to mitochondria dysfunction. Indeed, a similar accumulation of long-chain CARs in ischemic tissue has been reported elsewhere, and it is thought that this accumulation inhibits oxidative phosphorylation, induces mitochondrial membrane hyperpolarization, and stimulates the production of reactive oxygen species (ROS) (Liepinsh et al., 2016; Andrianova et al., 2020). LPCs and LPEs are the products of the phospholipase-induced hydrolysis of PCs and PEs (Xu et al., 2015; Law et al., 2019). These compounds play a role in cellular signal transduction, tumorigenesis, angiogenesis, immunity, atherosclerosis, cancer, and neuronal survival (Kordalewska et al., 2019). Previously, higher levels of LPCs have been associated with oxidative stress and pro-inflammatory effects (Kordalewska et al., 2019). Moreover, similar to the results obtained in this study, Xu et al. (2015) observed higher pretransplant levels of LPCs (LPC 16:0, LPC 18:0) and LPEs (LPE 16:0, LPE 18:0) in donors after circulatory death compared to donors after brain death. Conversely, Rao et al. found higher levels of LPC 18:0, LPC 26:6, LPE 16:0, and LPC 18:0, along with a corresponding reduction in LPC 20:0, LPC 20:4, LPE 22:0, and LPE 24:6, 24 h after IR-induced acute kidney injury (Rao et al., 2016). The inconclusive results obtained in recent studies may be related to the complexity of the enzymatic cascade involved in LPC metabolism (Law et al., 2019).

Chemometric analysis revealed significant differences between the SCS, HMP, and NEVKP preservation methods, with further in-depth analysis demonstrating that the method’s preservation temperature has a greater impact on the lipidomic profile than its mechanical character. Higher levels of CARs, PCs, ether-linked PCs, ether-linked PEs, phosphatidylinositols (PIs), TGs, most LPCs and LPEs, and longer-chain PEs were observed in the hypothermic group, while higher levels of ceramides (Cer), PSs, and shorter-chain PEs were observed in the normothermic group. Numerous SMs differentiated the preservation methods, but no dominant trend was observed. As mentioned above, increased levels of long-chain CARs may be related to mitochondria dysfunction and increased production of ROS (Liepinsh et al., 2016; Andrianova et al., 2020), and higher levels of LPCs may be associated with oxidative stress and pro-inflammatory effects (Kordalewska et al., 2019). Moreover, higher levels of LPEs were observed in DCD livers compared to livers from donors after brain death (Xu et al., 2015), and increased LPE levels were observed in CKD progression (Kordalewska et al., 2019). Rao et al. (2016) and Solati et al. (2018) have reported elevated levels of PCs in mice and rat ischemia/reperfusion (I/R) models. Similar to our results, they observed increases in PC 34:1, PC 34:2, PC 36:1, PC 36:2, PC 36:3, PC 36:4, PC 38:3, PC 38:4, PC 38:5, PC 38:6, PC 40:4 and PC 40:5. Additionally, Solati et al. reported increased levels of PIs 6 h and 12 h after I/R, while in this study higher levels of PI 36:2 and PI 38:4 were observed in the hypothermic preservation group (Solati et al., 2018). Ether-linked PCs and ether-linked PE were present at higher levels in the hypothermic group compared to the normothermic group. In Rao et al.’s study, significantly elevated levels of a small number of ether-linked phospholipids were observed 6 h after renal I/R (vs. control animals); notably, levels of these ether phospholipids were correlated with plasma creatinine. However, 24 h after I/R, only PC O-38:1 was still elevated, while ether-linked PEs were reduced at this later time point (Rao et al., 2016). Ether lipids differ from their diacyl counterparts, allowing them to contribute unique structural characteristics to biological membranes. Moreover, previous findings suggest that ether lipids play a role in various biological processes, including cell differentiation, cellular signalling, and oxidative-stress reduction (Dean and Lodhi, 2018). While alterations of ether lipids have been reported in several disorders, including neurodegenerative disease, cancer, and metabolic disorders (Rao et al., 2016; Dean and Lodhi, 2018), the molecular mechanism underlying their role in these pathologies remains unclear. Higher levels of three TGs (TG 44:1, TG 56:6, TG 56:7) and three longer-chain PEs (PE 38:4, PE 38:5, PE 40:4) were also observed in the hypothermic group. Similar elevations were reported by Afshinnia et al. (2018) in their study of lipid profiles in CKD. Their results indicated that higher quantities of long polyunsaturated lipids are related to CKD progression. Elsewhere, Solati et al. (2018) observed an increase in longer-chain PEs and a reduction in shorter-chain PEs 24 h after I/R, which aligns with the trend observed for the hypothermic group in our study. The alterations described above suggest the NEVKP method’s beneficial effects with respect to kidney grafts. The lower accumulation of lipids in NEVKP kidneys, especially those with pro-inflammatory properties, results in improved graft function following NEVKP compared to hypothermic preservation methods. Additionally, the reductions of CAR 12:0 and CAR 14:1 across perfusion time points may also be indicative of NEVKP’s beneficial effects.

A much smaller number of compounds differentiating the HMP, NEVKP, and SCS methods were identified post-perfusion (vs. during preservation). However, after reperfusion, the trend in changes was similar to that observed during perfusion, with most of the significant changes differentiating NEVKP from other hypothermic methods, rather than from HMP and SCS.

A comparison of the kidneys during reperfusion and on POD3 revealed more significant changes in the SCS group compared to the NEVKP group. In particular, the SCS group exhibited elevated levels of ether-linked PEs and PC P-38:4 on POD3. As noted above, Rao et al. reported higher levels of PC O-38:1, PE O-42:3, and PE O-40:4 6 h after I/R, but only PC O-38:1 remained elevated in I/R at 24 h. However, more research is required to investigate the molecular mechanisms underlying the role of ether lipids in kidney disease. Additionally, in the SCS group, lower levels of SMs were identified on POD3. Previous findings have shown an increase in SMs 24 h after I/R in animal models (Rao et al., 2016; Solati et al., 2018). In contrast, Zhao et al. (2014) observed lower SMs levels in an acute graft rejection group after transplantation, while Tofte et al. (2019) demonstrated that higher levels of specific SMs were associated with a lower risk of end-stage renal disease and all-cause mortality in type 1 diabetes. These findings indicate that lower levels of SMs may be related to impaired graft function. The results of this comparison (donor vs. POD3) provide further evidence that NEKVP enables improved graft function compared to SCS.

The last step of untargeted analysis entailed a comparison of the lipidomic profiles at baseline (donation) and reperfusion and at baseline and POD3. The largest number of statistically significant compounds was identified in the donor-POD3 comparison for the SCS group. While the trends in changes were similar for the SCS and NEVKP groups, the alterations were usually more noticeable in the SCS group. The observed alterations mainly concerned lipids described previously in this study. Higher levels of CARs, a few LPCs and LPEs, several PCs and PEs, and ether-linked PCs on POD3 were observed for both the SCS and NEVKP groups. However, more compounds among these lipid classes were statistically significant for the SCS group. As discussed above, the elevated levels of these lipids may be related to I/R injury, mitochondrial dysfunction, pro-inflammatory effect, and/or oxidative stress. Significant reductions in SM levels on POD3 were observed only for the SCS group. As previously noted, prior findings have shown a connection between reduced SM levels and acute graft rejection (Zhao et al., 2014).

Despite these promising results, this study has several limitations. The first limitation is the small sample sizes. However, since SPME enables multiple analyses over time, it is possible to acquire a large number of samples without requiring more animals. Another limitation was that the obtained results were not compared with routinely assessed clinical parameters; as such, the analysis was not as comprehensive as it might have otherwise been. A final limitation of this study design is the relatively short follow-up period of 3 days post-transplantation. Unfortunately, this interval was necessary, as the porcine experimental setup makes it difficult to investigate over a longer follow-up period of months, or even years.

## 5 Conclusion

SPME chemical biopsy followed by LC-MS/MS analysis enables the minimally invasive and repeated sampling of the same tissue. As such, this method was successfully applied to track alterations in a graft throughout the entire transplantation procedure, and to compare kidney lipidomic profiles during storage with different preservation methods. As a result, we observed that the preservation temperature has a more significant impact on the lipidomic profile of the kidney than the preservation method’s mechanical characteristics. Higher levels of CARs, PCs, ether-linked PCs, ether-linked PEs, PIs, TGs, most LPC and LPE, and longer-chain PEs were observed in the hypothermic preservation group, which may be related to I/R injury, mitochondrial dysfunction, pro-inflammatory effect, and oxidative stress. Hence, the obtained results suggest that the use of NEVKP can have a beneficial effect on graft function.

## Data Availability

The original contributions presented in the study are publicly available. This data can be found here: https://repod.icm.edu.pl/dataset.xhtml?persistentId=doi:10.18150/ATK9DA
