# Supplementary material for: The impact of normothermic and hypothermic preservation methods on kidney lipidome—comparative study using chemical biopsy with microextraction probes
Source: Front Mol Biosci. 2024 May 9;11:1341108. doi: 10.3389/fmolb.2024.1341108 (PMC11112113; doi:10.3389/fmolb.2024.1341108)
Supplement: Supplementary file 1 [file DataSheet1.PDF]

Supplementary Material

The impact of normothermic and hypothermic preservation methods on kidney lipidome – comparative study using chemical biopsy with microextraction probes

Natalia Warmuzińska[1], Kamil Łuczykowski[1], Iga Stryjak[1], Hernando Rosales-Solano[2], Peter Urbanellis[3], Janusz Pawliszyn[2], Markus Selzner[3,4], Barbara Bojko[1*]

[1]Department of Pharmacodynamics and Molecular Pharmacology, Faculty of Pharmacy, Nicolaus Copernicus University in Torun, Collegium Medicum in Bydgoszcz, Bydgoszcz, Poland
[2]Department of Chemistry, University of Waterloo, Waterloo, Ontario N2L 3G1, Canada
[3]Ajmera Transplant Center, Department of Surgery, Toronto General Hospital, University Health Network, Toronto, ON, Canada
[4]Department of Medicine, Toronto General Hospital, Toronto, ON, Canada

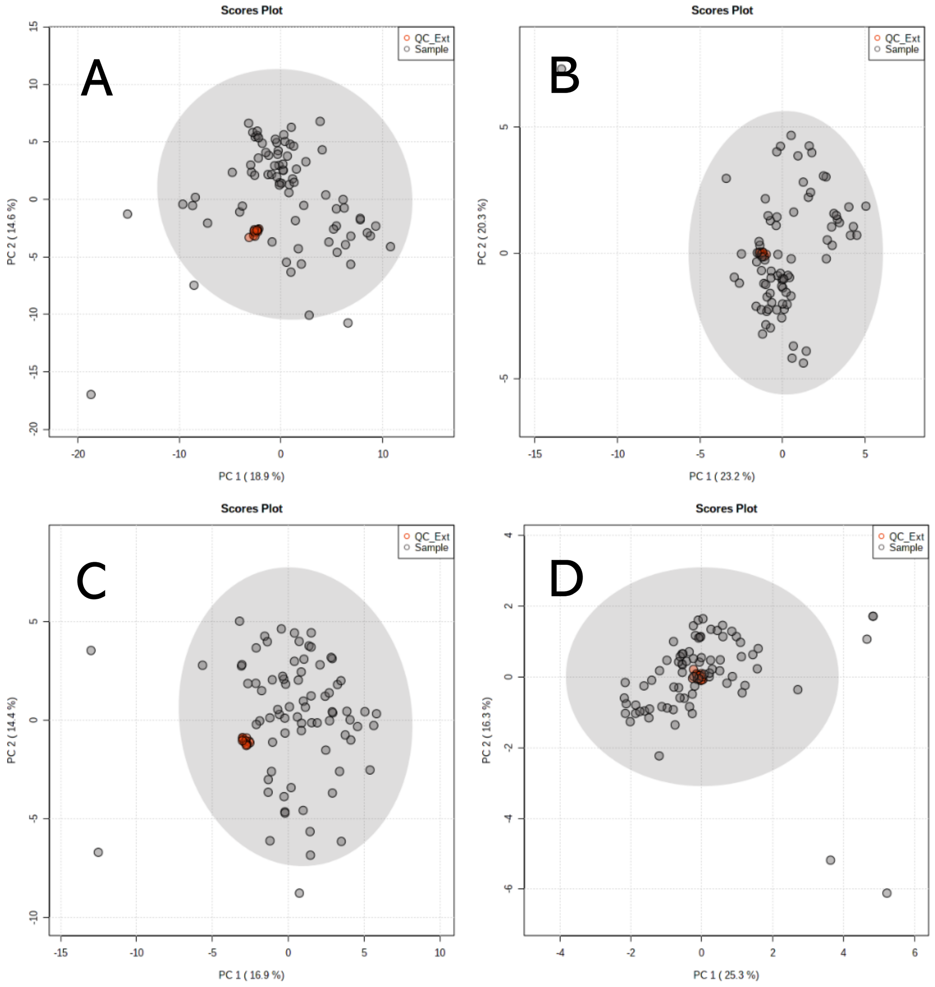

Fig. S1 Principal component analysis (PCA) plots of all analyzed samples and extraction quality control (QC_ext) samples. A- RP. positive ionization mode; B – RP. negative ionization mode; C- HILIC. positive ionization mode. D- HILIC. negative ionization mode.

Table S1. The list of lipid species identified using MS/MS mode.

Type of LC-HRMS analysis	number of compounds	Identified lipid species
HILIC neg HILIC pos RP neg RP pos	4	PE P-36:4; PE 34:2; PE 36:2; PC 34:1
HILIC pos RP neg RP pos	5	PC 38:5; PC 36:2; PC 36:4; PE 36:3; PC 36:3
HILIC neg RP neg RP pos	8	PE P-38:5; PE 38:4; PE 34:1; PE P-38:4; SM 34:1;O2; PE P-34:1; PC 34:2; PI 36:2
HILIC neg HILIC pos RP neg	3	LPE 20:4; PE 36:4; PE 38:5
RP neg RP pos	15	SM 42:3;O2; LPC 18:1; SM 42:1;O2; SM 42:2;O2; PC 36:1; PE P-36:1; SM 38:1;O2; LPC 18:0; LPC 16:0; PS 36:2; SM 40:1;O2; PS 36:1; LPC 18:2; PC 38:4; PE 36:1
HILIC pos RP pos	12	PC P-38:4; PC O-38:4; PC 36:5; SM 40:4;O2; PC 38:6; LPC 20:4; SM 44:5;O2; SM 36:4;O2; PC 40:5; LPC 18:3; SM 43:4;O2; PC 38:7
HILIC neg RP pos	5	PE P-40:4; PE P-34:2; PE P-34:4; PE P-38:6; PE P-40:5
HILIC neg RP neg	1	PI 38:4
RP pos	34	PE P-38:7; PC 35:1; PC P-34:2; SM 41:2;O2; PC O-36:4; Cer 42:1;O2; DG 38:4; TG 56:6; PC O-32:0; PC 33:1; PC 40:4; CE 18:2; PC 35:3; SM 37:1;O2; PC P-36:4; PC O-34:2; PE O-38:6; CE 20:4; PC O-34:0; SM 34:0;O2; PE 40:4; SM 36:1;O2; DG 38:5; SM 35:1;O2; Cer 42:2;O2; PC 38:3; Cer 41:1;O2; PC 32:1; Cer 40:1;O2; TG 56:7; Cer 38:1;O2; SM 39:1;O2; TG 44:1; MG 18:2
RP neg	4	Cer 34:1;O2; PC 32:0; PC 34:0; PS 38:4

HILIC pos	28	PC 37:4; SM 39:4;O2; PC 40:6; PE O-18:2; PC 21:4; LPE P-16:0; SM 42:5;O2; SM 42:4;O2; PC P-40:7; SM 35:4;O2; CAR 14:0; DG 28:2; PC 32:3; CAR 14:1; SM 36:3;O2; PC 40:8; CAR 14:2; PC 42:9; PS 38:5; SM 41:4;O2; CAR 12:0; SM 38:5;O2; SM 44:4;O2; CAR 18:2; PC P-38:7; SPB 17:0;O3; LPC 15:0; PC 35:6
HILIC neg	9	PE 38:6; PE P-40:6; PE 35:2; LPE 18:0; PI 34:1; LPE 18:2; LPE 18:1; PE P-36:5; LPE 22:4

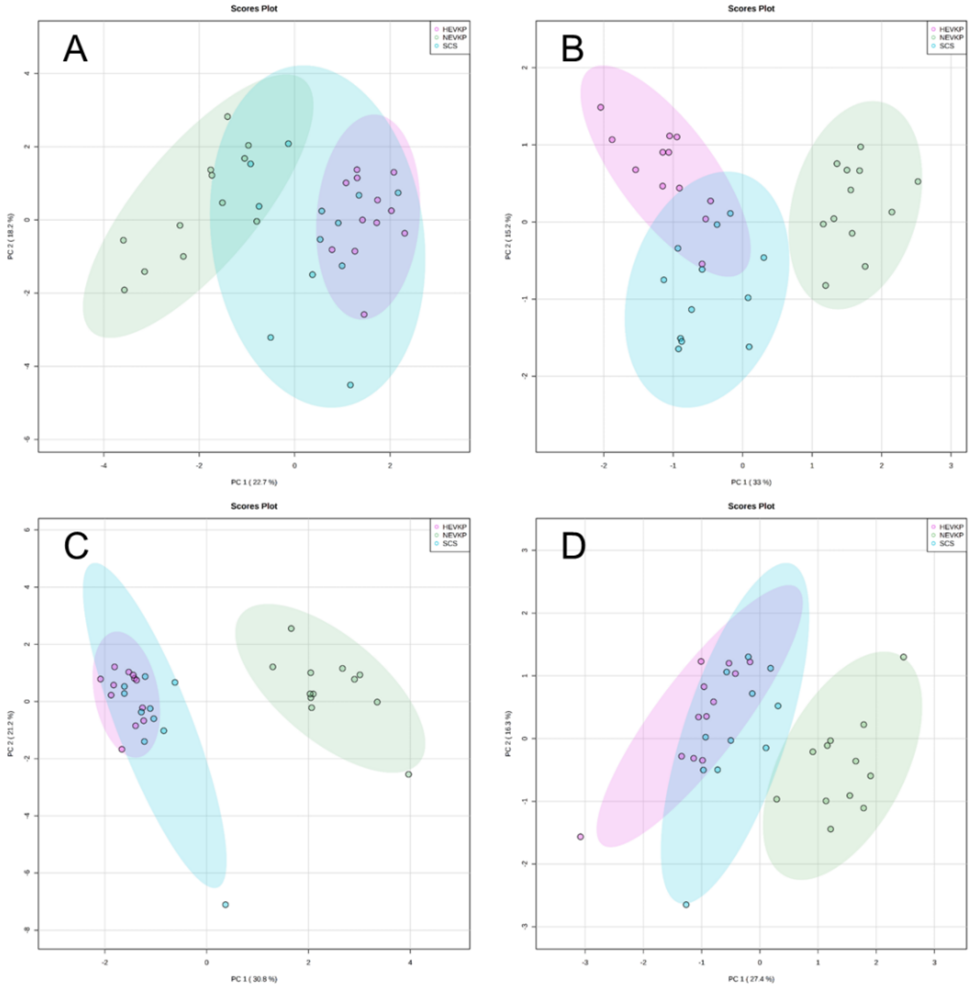

Fig. S2 Score plots (PCA) showing separation between different types of kidney preservation. HILIC analyses in positive (A) and negative (B) ionization modes and RP analyses in positive (C) and negative (D) ionization modes.

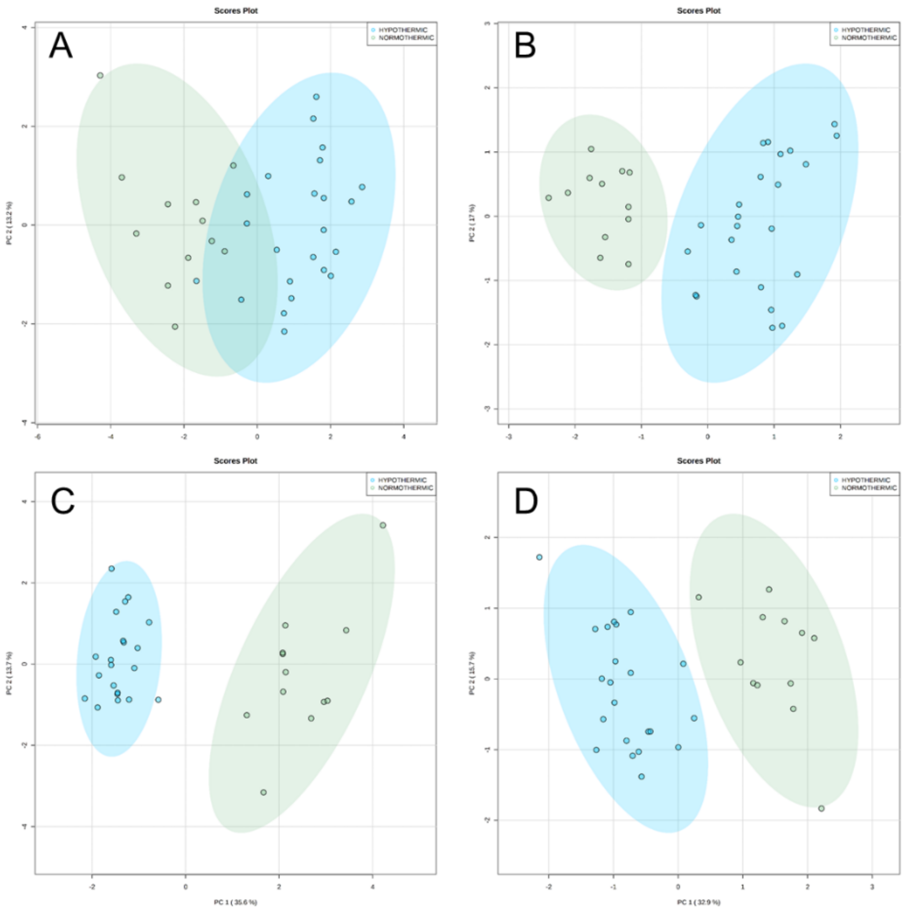

Fig. S3 Score plots (PCA) showing separation between normothermic and hypothermic preservation methods. HILIC analyses in positive (A) and negative (B) ionization modes and RP analyses in positive (C) and negative (D) ionization modes.

Table S2. The list of compounds that differentiated NEVKP, HMP, and SCS group during perfusion.

Name	Molecular Weight	RT [min]	FDR adjusted p-value	VIP
CAR 12:0	343.2723	8.19 (H)	0.0166	1.0978
CAR 14:1	369.2879	8.00 (H)	0.0023	1.2482
CAR 14:2	367.2723	8.07 (H)	0.00007	1.4621
CAR 18:2	423.3349	7.83 (H)	0.017	<1.0
CE 18:2	648.5845	13.96	0.00002	2.2419

CE 20:4	672.5845	13.88	0.004	<1.0
Cer 34:1;O2	537.5121	9.21	0.00001	1.5089
Cer 38:1;O2	593.5747	10.86	0.00004	<1.0
Cer 40:1;O2	621.606	11.44	0.00002	<1.0
Cer 41:1;O2	635.6216	11.70	0.0004	<1.0
Cer 42:1;O2	649.6373	11.91	0.00002	<1.0
Cer 42:2;O2	647.6216	11.48	0.00002	<1.0
DG 28:2	508.4128	1.40 (H)	>0.05	1.8271
DG 38:4	644.5379	11.20	0.0004	<1.0
DG 38:5	642.5223	10.70	>0.05	1.4463
LPC 15:0	481.3168	9.01 (H)	0.0532	1.0966
LPC 18:0	523.3638	4.65	0.007	<1.0
LPC 18:1	521.3481	4.00	0.0016	<1.0
LPC 18:2	519.3325	3.76	0.0446	<1.0
LPC 20:4	543.3325	8.47 (H)	0.00007	1.32
LPE 18:1	479.3012	9.89 (H)	0.0062	<1.0
LPE 20:4	501.2855	9.71 (H)	0.0002	1.0298
LPE 22:4	529.3168	9.37 (H)	0.00001	1.3161
LPE P-16:0	437.2906	9.44 (H)	0.0069	1.3714
PC 21:4	571.3274	7.73 (H)	0.00002	1.9972
PC 32:0	733.5622	9.09	0.0012	1.2178
PC 32:1	731.5465	8.68	>0.05	1.0028
PC 33:1	745.5622	9.06	0.0001	<1.0
PC 34:0	761.5935	9.87	>0.05	1.1281
PC 34:1	759.5778	9.49	0.00001	<1.0
PC 34:2	757.5622	8.96	0.00001	<1.0
PC 36:1	787.6091	10.23	0.00007	<1.0
PC 36:2	785.5935	9.78	0	<1.0
PC 36:3	783.5778	8.95	0.0004	<1.0
PC 36:4	781.5622	6.8 (H)	0.0008	<1.0
PC 38:3	811.6091	10.06	0.0001	<1.0
PC 38:4	809.5935	9.44	0.0002	<1.0
PC 38:5	807.5778	8.98	0.00001	1.0578

PC 38:6	805.5622	6.74 (H)	0.0045	<1.0
PC 40:4	837.6248	10.33	0.00003	1.5863
PC 40:5	835.6091	6.74 (H)	0.0012	1.1539
PC 40:6	833.5935	6.72 (H)	0.0419	<1.0
PC 40:8	829.5622	6.74 (H)	0.0049	1.2917
PC 42:9	855.5778	6.68 (H)	0.0003	1.2953
PC O-38:4	795.6142	10.25	>0.05	1.5866
PC P-36:4	765.5672	9.39	0.0001	<1.0
PC P-38:4	793.5985	9.65	0.001	1.929
PC P-38:7	787.5516	6.70 (H)	0.0103	<1.0
PE 34:1	717.5309	9.45	0	1.6464
PE 34:2	715.5152	9.11	0	1.3767
PE 35:2	729.5309	7.24 (H)	0.011	1.3977
PE 36:1	745.5622	10.16	0.0001	<1.0
PE 36:2	743.5465	9.73	0.00001	<1.0
PE 36:3	741.5309	9.10	0.0004	1.4669
PE 36:4	739.5152	8.93	0.0003	1.325
PE 38:4	767.5465	7.07 (H)	0.0044	<1.0
PE 38:5	765.5309	9.12	0.0003	1.3479
PE 40:4	795.5778	10.42	0.0011	1.3142
PE O-18:2	477.2855	9.52 (H)	0.0028	1.0759
PE O-38:6	749.5359	9.71	0.0003	2.0622
PE P-34:1	701.5359	10.03	0.0162	<1.0
PE P-34:2	699.5203	9.57	0.0146	1.81
PE P-34:4	695.489	7.08 (H)	0.00001	1.0198
PE P-36:1	729.5672	10.70	>0.05	1.4895
PE P-36:4	723.5203	9.37	0.0002	1.4724
PE P-36:5	721.5046	7.04 (H)	0.0009	<1.0
PE P-38:4	751.5516	6.95 (H)	0.0006	1.2651
PE P-38:5	749.5359	6.96 (H)	0.00001	2.1539
PE P-38:6	747.5203	9.49	0.0002	1.357
PE P-38:7	745.5046	9.54	0.0003	<1.0
PE P-40:4	779.5829	6.93 (H)	0.0002	1.9236

PE P-40:5	777.5672	6.98 (H)	0.00003	2.2587
PE P-40:6	775.5516	6.91 (H)	0.0002	1.8385
PI 36:2	862.5571	8.19	0.0008	1.6546
PI 38:4	886.5571	7.03 (H)	0.0046	<1.0
PS 36:1	789.552	8.60	0.014	1.351
PS 36:2	787.5363	8.31	0.0353	<1.0
PS 38:5	809.5207	8.39 (H)	0.0229	1.0124
SM 34:0;O2	704.5832	8.62	0.0021	<1.0
SM 34:1;O2	702.5676	8.09	0.0006	1.6464
SM 35:1;O2	716.5832	8.74	0.0226	<1.0
SM 35:4;O2	710.5363	8.23 (H)	0.0196	<1.0
SM 36:3;O2	726.5676	7.94 (H)	0.0005	<1.0
SM 36:4;O2	724.5519	8.17 (H)	0.0097	<1.0
SM 38:1;O2	758.6302	10.00	0.00005	<1.0
SM 38:5;O2	750.5676	8.04 (H)	0.04	1.0147
SM 39:1;O2	772.6458	10.35	0.0003	<1.0
SM 39:4;O2	766.5989	8.07 (H)	0.0027	1.0214
SM 40:1;O2	786.6615	10.66	0.016	<1.0
SM 40:4;O2	780.6145	9.99	0.00009	<1.0
SM 41:2;O2	798.6615	10.41	0.00006	1.1097
SM 41:4;O2	794.6302	7.99 (H)	0.00003	<1.0
SM 42:1;O2	814.6928	11.09	0.049	<1.0
SM 42:2;O2	810.6615	10.72	0.0034	<1.0
SM 42:3;O2	810.6615	10.12	0.0256	1.4516
SM 42:4;O2	808.6458	7.98 (H)	0.0002	1.4343
SM 44:5;O2	834.6615	7.94 (H)	0.0334	<1.0
SPB 17:0;O3	303.2773	3.76 (H)	0.0142	1.0497
TG 44:1	748.6581	5.42	0.0036	<1.0
TG 56:6	906.7676	13.84	0.003	1.6641
TG 56:7	904.752	13.63	0.0007	1.6817

(H) indicates that the compound was identified with the use of the HILIC separation method; otherwise compound was identified with the use of the RP separation method

Table S3. The list of compounds that differentiated normothermic and hypothermic preservation methods during perfusion.

Name	Molecular Weight	RT [min]	FDR adjusted p-value	VIP
**CAR 12:0**	**343.2723**	**8.19 (H)**	**0.012998**	**1.5032**
**CAR 14:1**	**367.2723**	**8.00 (H)**	**0.0013804**	**1.922**
**CAR 14:2**	**369.2879**	**8.07 (H)**	**0.000016643**	**2.4572**
**CE 18:2**	**648.5845**	**13.96**	**0.00036187**	**1.4812**
CE 20:4	672.5845	13.88	0.0017392	1.7246
Cer 34:1;O2	537.5121	9.21	0.00000004863	2.2529
Cer 38:1;O2	593.5747	10.86	0.0000017624	1.5236
Cer 40:1;O2	621.6060	11.44	0.00000030131	1.4366
Cer 41:1;O2	635.6216	11.7	0.000038106	1.1275
Cer 42:1;O2	649.6373	11.91	0.000000094697	1.3617
Cer 42:2;O2	647.6216	11.48	0.0000012533	1.4212
DG 38:4	644.5379	11.2	0.00012417	1.6011
**LPC 18:0**	**523.3638**	**4.65**	**0.0066634**	**<1.0**
**LPC 18:1**	**521.3481**	**4.00**	**0.00098038**	**1.1696**
LPC 18:2	519.3325	3.76	0.017806	1.0582
**LPC 20:4**	**543.3325**	**8.47 (H)**	**0.00028469**	**1.5064**
LPE 18:1	479.3012	9.89 (H)	0.00088297	<1.0
**LPE 18:2**	**477.2855**	**9.98 (H)**	**0.043876**	**<1.0**
**LPE 20:4**	**501.2855**	**9.71 (H)**	**0.00085391**	**1.2297**
**LPE 22:4**	**529.3168**	**9.37 (H)**	**0.00000047936**	**3.3425**
**PC 21:4**	**571.3274**	**7.73 (H)**	**0.0000061078**	**2.8498**
PC 33:1	745.5622	9.06	0.0000024173	1.1984
**PC 34:1**	**759.5778**	**9.49**	**0.000000094697**	**1.0634**
**PC 34:2**	**757.5622**	**8.96**	**0.00000043706**	**<1.0**
**PC 36:1**	**787.6091**	**10.23**	**0.000066651**	**<1.0**
**PC 36:2**	**785.5935**	**9.78**	**0.00000021044**	**1.3105**
**PC 36:3**	**783.5778**	**8.95**	**0.000077031**	**1.0044**
**PC 36:4**	**781.5622**	**8.97**	**0.000000094697**	**1.0376**
**PC 36:5**	**779.5465**	**6.84 (H)**	**0.037041**	**<1.0**

**PC 38:3**	**811.6091**	**10.06**	**0.0000080062**	**1.1074**
**PC 38:4**	**809.5935**	**9.44**	**0.00022188**	**<1.0**
**PC 38:5**	**807.5778**	**8.98**	**0.000016461**	**1.1869**
**PC 38:6**	**805.5622**	**8.62**	**0.00000021044**	**1.4147**
**PC 38:7**	**803.5465**	**6.76 (H)**	**0.04374**	**<1.0**
**PC 40:4**	**837.6248**	**10.33**	**0.004057**	**<1.0**
**PC 40:5**	**835.6091**	**9.93**	**0.0000032907**	**1.3286**
PC 40:8	829.5622	6.74 (H)	0.037529	<1.0
**PC P-36:4**	**765.5672**	**9.39**	**0.000054189**	**1.0278**
**PC P-38:4**	**793.5985**	**9.65**	**0.012435**	**1.0888**
**PC P-38:7**	**787.5516**	**6.70 (H)**	**0.04374**	**<1.0**
PE 34:1	717.5309	9.45	0.00000004863	2.8384
PE 34:2	715.5152	9.11	0.00000004863	2.2595
PE 35:2	729.5309	7.24 (H)	0.010817	1.3899
PE 36:1	745.5622	10.16	0.000000072946	1.6152
PE 36:2	743.5465	7.23 (H)	0.00092477	<1.0
PE 36:4	739.5152	7.12 (H)	0.00017714	1.0423
**PE 38:4**	**767.5465**	**9.92**	**0.00000059975**	**<1.0**
**PE 38:5**	**765.5309**	**7.07 (H)**	**0.033085**	**1.942**
**PE 40:4**	**795.5778**	**10.42**	**0.0060529**	**<1.0**
PE O-18:2	477.2855	9.52 (H)	0.025049	<1.0
**PE O-38:6**	**749.5359**	**9.71**	**0.0035824**	**<1.0**
**PE P-34:4**	**695.4890**	**7.08 (H)**	**0.00015334**	**1.0254**
PE P-36:1	729.5672	10.7	>0.05	1.1297
**PE P-36:4**	**723.5203**	**7.00 (H)**	**0.00041293**	**<1.0**
**PE P-38:5**	**749.5359**	**9.71**	**0.00049051**	**1.0787**
**PE P-38:6**	**747.5203**	**9.49**	**0.00030555**	**1.0599**
**PE P-38:7**	**745.5046**	**9.54**	**0.000045489**	**<1.0**
**PE P-40:5**	**777.5672**	**10.41**	**0.019295**	**<1.0**
**PI 36:2**	**862.5571**	**8.19**	**0.000045489**	**1.2293**
**PI 38:4**	**886.5571**	**8.04**	**0.018924**	**0.008516**
PS 36:1	789.5520	8.6	0.0042238	1.0546
PS 36:2	787.5363	8.31	0.0095296	0.0035736

SM 34:0;O2	704.5832	8.62	0.0013125	<1.0
SM 34:1;O2	702.5676	8.28 (H)	0.01054	<1.0
**SM 36:1;O2**	**730.5989**	9.19	**0.047821**	**<1.0**
SM 36:3;O2	726.5676	7.94 (H)	0.00096509	1.3935
SM 36:4;O2	724.5519	8.17 (H)	0.010047	2.5622
**SM 38:1;O2**	**758.6302**	**10.00**	**0.000000094697**	**1.1772**
**SM 39:1;O2**	**772.6458**	**10.35**	**0.000013937**	**1.1867**
**SM 39:4;O2**	**766.5989**	**8.07 (H)**	**0.009437**	**2.6194**
**SM 40:1;O2**	**786.6615**	**10.66**	**0.0035824**	**<1.0**
**SM 40:4;O2**	**780.6145**	**9.99**	**0.00000043706**	**1.2831**
**SM 41:2;O2**	**798.6615**	**10.41**	**0.00042726**	**1.0297**
SM 41:4;O2	794.6302	7.99 (H)	0.0000061078	1.9875
**SM 42:2;O2**	**810.6615**	**10.72**	**0.00049051**	**<1.0**
SM 42:4;O2	808.6458	7.98 (H)	>0.05	1.0229
SM 44:5;O2	834.6615	7.94 (H)	0.037041	1.8495
SPB 17:0;O3	303.2773	3.76 (H)	0.012998	1.9689
**TG 44:1**	**748.6581**	**5.42**	**0.0013125**	**1.673**
**TG 56:6**	**906.7676**	**13.84**	**0.0017392**	**1.7051**
**TG 56:7**	**904.7519**	**13.634**	**0.00030555**	**2.1605**

Compounds with a higher level in hypothermic preservation methods are highlighted in bold. (H) indicates that the compound was identified with the use of the HILIC separation method; otherwise compound was identified with the use of the RP separation method

Table S4. The list of compounds that changed between time points throughout kidney perfusion.

**Name**	**Molecular weight**	**RT [min]**	**FDR adjusted p-value**
	**HMP**		
LPE P-16:0	437.2906	9.44 (H)	0.04206
LPC 18:3	517.3168	8.66 (H)	0.02929
PC 36:5	779.5465	6.84 (H)	0.04206
PC 36:2	785.5935	6.82 (H)	0.04206
PE P-38:6	747.5203	6.99 (H)	0.04206
PC 34:1	759.5778	6.84 (H)	0.02929
PE 34:1	717.5309	9.45	0.04206

PE P-38:7	745.5046	9.54	0.04206
PC 38:7	803.5465	8.97	0.04206
PC 38:6	805.5622	8.62	0.02929
SM 43:4;O2	822.6615	10.96	0.04206
PE 36:2	743.5465	9.73	0.04206
PE 36:1	745.5622	10.16	0.04206
SM 40:1;O2	786.6615	10.51	0.02929
PI 36:2	862.5571	7.98	0.04206
SM 42:1;O2	814.6928	11.09	0.04206
**NEVKP**			
CAR 12:0	343.2723	8.19 (H)	0.04206
CAR 14:1	369.2879	8.00 (H)	0.04206
TG 56:7	904.7519	13.63	0.04206

(H) indicates that the compound was identified with the use of the HILIC separation method; otherwise compound was identified with the use of the RP separation method

Table S5 The list of compounds that differentiate reperfusion and POD3 time points for SCS and NEVKP groups.

Name	Molecular weight	RT [min]	FDR adjusted p-value
**SCS**			
**PC 32:0**	**733.5622**	9.09	**0.02524**
**PC 32:1**	**731.5465**	8.68	**0.0136**
PC 35:6	763.5152	7.13 (H)	0.01885
PC 38:5	807.5778	6.78 (H)	0.01396
**PC P-38:4**	**793.5985**	9.65	**0.03351**
PE 36:3	741.5309	7.13 (H)	0.01885
PE 36:4	739.5152	7.12 (H)	0.02524
**PE P-38:4**	**751.5516**	10.16	**0.00537**
**PE P-40:4**	**779.5829**	6.93 (H)	**0.01885**
**PE P-40:5**	**777.5672**	10.41	**0.03351**
PS 36:2	785.5935	8.31	0.02524
SM 36:4;O2	724.5519	8.17 (H)	0.04409
SM 38:1;O2	758.6302	10.00	0.03351
SM 38:5;O2	750.5676	8.04 (H)	0.04409
SM 39:4;O2	766.5989	8.07 (H)	0.04409

SM 40:1;O2	786.6615	10.66	0.01885
SM 41:4;O2	794.6302	7.99 (H)	0.01396
SM 42:1;O2	814.6928	11.09	0.00745
SM 42:5;O2	806.6302	7.98 (H)	0.02524
SM 44:4;O2	836.6771	7.93 (H)	0.01395
SM 44:5;O2	834.6615	7.94 (H)	0.01396
	**NEVKP**		
**Cer 42:1;O2**	**649.6373**	11.91	**0.04409**
**PC 32:1**	**731.5465**	8.681	**0.02524**
**PC 36:1**	**787.6091**	10.23	**0.03351**

Compounds with a higher level on POD3 are highlighted in bold. (H) indicates that the compound was identified with the use of the HILIC separation method; otherwise compound was identified with the use of the RP separation method

Table S6 The list of statistically significant compounds in comparisons donor versus reperfusion for NEVKP, SCS, and HMP groups and donor versus POD3 for NEVKP and SCS groups.

			**SCS**		**NEVKP**		**HEVKP**
**Name**	**Molecular weight**	**RT [min]**	**donor / reperf**	**donor / POD3**	**donor / reperf**	**donor / POD3**	**donor / reperf**
CAR 12:0	343.2723	8.19 (H)	-1.0269	-2.3863*	-0.75457	-1.9376	-0.72092*
CAR 14:0	371.3036	8.02 (H)	-0.99743	-1.4458*	-0.14411	-1.4335	-0.53976
CAR 14:1	369.2879	8.00 (H)	-1.8512	-3.2537*	-1.4426	-2.6111*	-1.579*
CAR 14:2	367.2723	8.07 (H)	-1.9052	-3.8205*	-1.5529	-2.6519*	-1.843
Cer 34:1;O2	537.5121	9.21	-0.40997	-3.032	-0.10856	-1.0166*	-0.51166
Cer 38:1;O2	593.5747	10.86	-0.99705	-2.5056*	0.15087	-0.8167	-0.1308
DG 38:4	644.5379	11.2	0.80067	0.85205	1.203*	0.82122	0.66206
LPC 18:0	523.3638	4.65	1.4609	-0.52977	2.0926*	1.2109	1.1624
LPE 18:1	479.3012	9.89 (H)	-0.63591	-1.9022	-0.013194	-0.70323*	-0.2593
LPE 18:2	477.2855	9.98 (H)	-1.5412	-2.2378*	-0.15548	-1.7369*	-0.37405
LPE 20:4	501.2855	3.76	-0.27498	-3.10097*	1.1081	-1.187	-0.55835
LPE 22:4	529.3168	9.37 (H)	1.5896	0.69982	2.8471*	1.0904	1.3292
PC 21:4	529.2805	7.73 (H)	-1.2417	-2.895*	2.2114	-1.4275	-0.58296

PC 33:1	745.5622	9.06	-0.40149	-0.84172*	-0.30252	-0.81399*	-0.16674
PC 34:1	759.5778	6.84 (H)	0.01432	0.58265*	-0.15719	0.27859	-0.1077
PC 34:1	759.5778	9.49	0.45528	0.46389	0.38095	0.69702*	0.16379
PC 34:2	759.5778	6.95 (H)	0.2117	1.2472*	0.59748*	0.35358	0.20555
PC 36:1	787.6091	10.23	0.84198*	0.69536	0.84269*	0.80845*	0.41556
PC 36:2	785.5935	9.78	0.4727*	0.16546	0.68802*	-0.059386	-0.17535
PC 36:3	783.5778	8.95	0.34175	0.38267	0.63001*	0.26322	0.012774
PC 36:4	781.5622	6.80 (H)	0.37439	1.1162*	0.10911	0.88437*	0.017423
PC 36:5	779.5465	6.84 (H)	-0.072387	0.972*	-0.056695	0.50315	-0.14297
PC 38:4	809.5935	9.44	0.84313*	0.68637	0.81954*	0.8057*	0.40188
PC 38:5	807.5778	8.98	0.61825	0.91831*	0.21079	0.59955	-0.12913
PC 38:6	805.5622	8.62	0.25825	1.2624*	0.79813	0.96236*	-0.33124
PC 38:7	803.5465	6.76 (H)	0.077109	0.82746*	0.23124	0.64013*	-0.16289
PC 40:5	835.6091	6.74 (H)	0.73801	0.73708	0.61389	0.86268*	0.32582
PC 40:5	835.6091	9.93	0.96415*	0.67956	1.0829*	0.75059	0.25282
PC 40:6	833.5935	6.72 (H)	0.27463	0.89181*	0.13454	0.47191	0.29794
PC 42:9	855.5778	6.68 (H)	0.90879	0.92674	0.80355	0.9963*	0.5287
PC O-34:0	747.6142	10.48	-1.5735	-2.7817*	-1.5612	-3.3872*	-1.1642
PC O-34:2	743.5829	9.47	-0.46445	-1.2475*	-0.16527	-1.269	-0.50044
PC O-38:4	795.6142	10.25	-0.31996	-2.6007*	-0.38926	-2.9146*	-0.50296
PC P-38:7	787.5516	6.70 (H)	0.18991	0.65357*	0.15964	0.78866	-0.44508
PC P-40:7	815.5829	6.72 (H)	0.37719	2.0072*	0.22381	1.3804*	0.43299
PE 34:1	717.5309	9.45	-0.96572	-0.20002	-0.66938*	-0.67849	0.046835
PE 36:1	745.5622	10.16	-0.51095	-1.2379*	-0.3879	-1.1418*	-0.34438
PE 36:2	743.5465	9.73	-0.36957	-0.4227	-0.31647	-0.53943*	-0.20688
PE 38:5	765.5309	7.07 (H)	0.059547	0.90129*	0.15016	0.42284	0.098406
PE 38:6	763.5152	7.09 (H)	-0.17372	1.4787*	0.12979	0.44272	0.10214
PE O-18:2	477.2855	9.52 (H)	-1.0905	-1.932	-0.56747	-1.1151*	-0.83822
PE P-36:1	729.5672	10.7	-0.7646	-1.9321*	-0.76332	-1.9386*	-0.59635
PE P-36:4	723.5203	9.37	0.26296	0.35409	0.51307*	0.52871	-0.044015
PE P-38:5	749.5359	9.71	0.82991*	0.58902	-0.056433	0.6526	-0.20911
PI 38:4	886.5571	7.03 (H)	0.64879	0.80067	0.01842	0.17178	0.59439*
PS 36:1	789.552	8.6	0.060431	1.3737*	-0.64782	0.6261	-0.19287

PS 38:4	811.5363	8.12	0.011394	1.3719*	-0.71544	0.37107	-0.082197
SM 35:1;O2	716.5832	8.74	-0.11284	1.1341*	0.51365	0.46723	0.076761
SM 36:1;O2	730.5989	9.19	0.40029	1.037*	0.3062	0.52821	0.36417
SM 38:1;O2	758.6302	10.00	0.026363	1.0932*	0.39029	0.62661	-0.17324
SM 39:1;O2	772.6458	10.35	0.02087	1.8054*	0.66539	1.074	-0.11889
SM 40:1;O2	786.6615	10.66	0.020008	0.86254*	-0.0097978	0.21608	0.13182
SM 40:4;O2	780.6145	9.99	0.069289	1.3723*	0.42525	0.56387*	-0.14442
SM 42:1;O2	814.6928	11.09	-0.69296	0.36006	0.034172	-0.1674	-0.36465*
SM 42:4;O2	808.6458	7.98 (H)	0.49007	1.3599*	0.096101	0.7516	-0.57964
SM 42:5;O2	806.6302	7.98 (H)	-0.27168	1.2378	-1.1471*	-0.30861	-0.21303
SM 43:4;O2	822.6615	7.96 (H)	0.6791	1.1387*	0.31265	0.58048	0.16728
SM 44:4;O2	836.6771	7.93 (H)	-0.79871	1.2099*	-0.18159	-0.59287	0.12269
SM 44:5;O2	834.6615	7.94 (H)	-0.54956	0.90684*	-0.18159	-0.11173	-0.04964

Values are reported as a log2(FC); FC- fold change. FCs are calculated as the ratios between two group means. (H) indicates that the compound was identified with the use of the HILIC separation method; otherwise compound was identified with the use of the RP separation method. * is p value < 0.05
